# Pharmacovigilance study of GLP-1 receptor agonists for metabolic and nutritional adverse events

**DOI:** 10.3389/fphar.2024.1416985

**Published:** 2024-07-08

**Authors:** Long He, Qiuyu Li, Yongfeng Yang, Jiahao Li, Wei Luo, Yilan Huang, Xiaoyan Zhong

**Affiliations:** ^1^ Department of Pharmacy, The Affiliated Hospital of Southwest Medical University, Luzhou, China; ^2^ School of Pharmacy, Southwest Medical University, Luzhou, China

**Keywords:** GLP-1 receptor agonists, FAERS, adverse events, metabolism and nutrition disorders, pharmacovigilance

## Abstract

**Aims:** Glucagon-like peptide-1 receptor agonists (GLP-1 RAs) are employed extensively in the management of type 2 diabetes and obesity. However, there is a paucity of real-world data on their safety and tolerability for metabolic and nutritional adverse events in large sample populations. This study aimed to analyse the metabolic and nutritional safety signatures of different GLP-1 RAs by exploring the Food and Drug Administration (FDA) Adverse Event Reporting System (FAERS).

**Methods:** AEs data were extracted from the FDA Adverse Event Reporting System database for each GLP-1 RA from the time of its launch until the second quarter of 2023. The reported odds ratio (ROR), proportional reporting ratio (PRR), Empirical Bayesian Geometric Mean and Bayesian Confidence Propagation Neural Network were employed to identify AE signals.

**Results:** A system organ class of metabolism and nutrition disorders was employed to filter AE reports, resulting in the identification of 10,450 reports for exenatide, 2,860 reports for liraglutide, 240 reports for albiglutide, 4,847 reports for dulaglutide, 2,905 reports for semaglutide, 1,089 reports for tirzepatide, and 13 reports for lixisenatide. Semaglutide (ROR, 3.34; 95%CI, 3.22), liraglutide (ROR, 2.78; 95%CI, 2.69), and exenatide (ROR, 2.15; 95%CI, 2.11) were associated with metabolism and nutrition disorders. The number of AE signals detected were as follows: albiglutide (n = 1), lixisenatide (n = 2), tirzepatide (n = 11), exenatide (n = 12), liraglutide (n = 16), semaglutide (n = 20), dulaglutide (n = 22). Dehydration was the most frequent AE contributing to serious outcomes for liraglutide (n = 318, 23.93%), dulaglutide (n = 434, 20.90%), semaglutide (n = 370, 25.10%) and tirzepatide (n = 70, 32.86%). The time to onset (TTO) of AE was statistically different between exenatide and the other GLP-1 RAs (*p* < 0.001), and the Weibull parameters for dehydration for liraglutide, dulaglutide, and semaglutide analyses all showed an early failure-type profile.

**Conclusion:** Our study suggests that exenatide, liraglutide, and semaglutide are more susceptible to metabolic and nutritional AEs than other GLP-1 RAs. Liraglutide, dulaglutide, semaglutide, and tirzepaptide’s potential to induce dehydration, necessitates special attention. Despite certain deficiencies, GLP-1 RAs have considerable potential for the treatment of eating disorders.

## 1 Introduction

Obesity and diabetes constitute formidable challenge to public health, affecting upwards of one billion individuals worldwide (Bailey et al., 2024). The International Diabetes Federation (IDF) Diabetes Atlas reports that the prevalence of diabetes among individuals aged 20–79 stood at 10.5% in 2021, encompassing approximately 536.6 million people, with projections suggesting an escalation to 12.2% (783.2 million people) by 2045. Global healthcare expenditures related to diabetes in 2021 were estimated at 966 billion USD, and forecasted increase to 1,054 billion USD by 2045 (Sun et al., 2022). Glucagon-like peptide-1 receptor agonists (GLP-1 RAs) have emerged as a key therapeutic option in the management of type 2 diabetes mellitus (T2DM), owing to their efficacy in lowering blood glucose, in addition to providing cardiovascular and renal benefits, and ameliorating metabolic syndrome, as supported by a wealth of evidence-based research (Davies et al., 2022). GLP-1RAs increase satiety, reduce food intake, and delay gastric emptying (Drucker, 2018; Novikoff and Müller, 2024). These effects have further expanded the utility of GLP-1RAs in weight loss in obese patients (Tan et al., 2022; Bailey et al., 2023; Camilleri and Acosta, 2024). So far, the United States Food and Drug Administration (FDA) has approved liraglutide in December 2014 and semaglutide in 2020 for the treatment of obesity.

Some concerns have been raised among healthcare professionals over the safety profiles of GLP-1 Ras. There have been reports of suicidal or self-injurious behaviors (SSIBs) to the European Medicines Agency (EMA), although pharmacovigilance reviews have reported no direct link between GLP-1 RAs and SSIBs (Chen et al., 2023; Chen et al., 2024; Zhou et al., 2024). Increased risks of thyroid cancer and cholecystitis have been reported (He et al., 2022; Bezin et al., 2023).

Recent research has focused on the potential use of GLP-1RAs in treating eating disorders, specifically binge eating (McElroy et al., 2018; Aoun et al., 2024; Balantekin et al., 2024). Despite the appetite-suppressing effects of GLP-1 RAs, concerns have been raised regarding their impact on eating disorder symptoms and management (Bartel et al., 2024; Richards and Khalsa, 2024). Pharmacovigilance studies on the metabolic and nutritional adverse events of GLP-1 RAs are still ongoing. The aims of the present study was to identify potential signals of adverse metabolic and nutritional reactions associated with GLP-1 RA use. By doing so, we hope to provide valuable insights into the application and research of GLP-1 RAs in managing eating disorders and other metabolic conditions.

## 2 Materials and methods

### 2.1 Data sources

**FIGURE 1:**
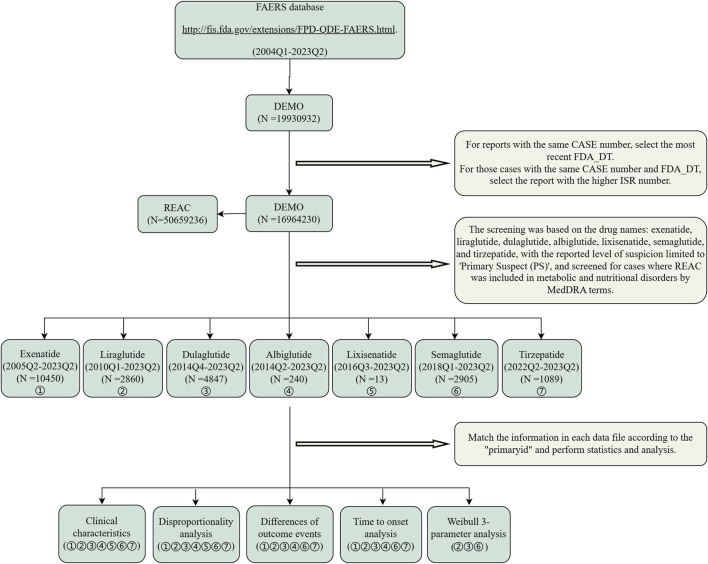
Flow chart of data extraction and analysis. A detailed description of the flow chart for data extraction and analysis of adverse events for GLP-1 RAs for metabolism and nutrition disorders in the United States Food and Drug Administration Adverse Event Reporting System (FAERS).

The data extraction and analysis procedure are shown in Figure 1. This retrospective pharmacovigilance study utilized data from the FDA Adverse Event Reporting System (FAERS) (https://fis.fda.gov/extensions/FPD-QDE-FAERS/FPD-QDE-FAERS.html), which consists primarily of adverse event reports reported by health professionals, pharmaceutical manufacturers, attorneys, and individual patients, reports of medication errors with medication administration, and product quality complaints. The database consists of seven data files: patient demographics (DEMO), drug information (DRUG), adverse event information (REAC), patient outcome information (OUTC), reported source information (RPSR), drug treatment date information (THER), and indication for drug (INDI) (Shu et al., 2022). Adverse events were classified using standardized Medical Dictionary for Regulatory Activities (MedDRA) terms for consistency and global recognition. GLP-1 RAs were analyzed as primary suspected (PS) from Q1 2004 to Q2 2023, with duplicate reports removed using recommended methods (Zhang et al., 2024). Data were collected for 16,964,230 patients and included 50,659,236 AE reports, focusing on AEs related to the system organ class (SOC) of metabolism and nutrition disorders. It is worth noting that the serious outcomes include death, life-threatening events, hospitalization, disability, congenital anomalies, and other serious consequences.

### 2.2 Disproportionate analysis

Disproportionality analysis is a commonly used method for post-marketing surveillance of adverse reaction signals in pharmaceuticals, which includes reporting ratio of ratios (ROR), proportional reporting ratios (PRR), Empirical Bayesian Geometric Mean (EBGM), and Bayesian Confidence Propagation Neural Network (BCPNN). Several studies have shown that Bayesian methods are superior to frequency counting approaches such as PRR, but EBGM and BCPNN are less sensitive and tend to detect fewer signals than ROR and PRR. The study used four approaches to detect adverse reaction signals (Evans et al., 2001; Szarfman et al., 2002). The system organ class (SOC), high level term (HLT) and preferred term (PT) tiers of the target drugs were detected, simultaneously fulfilling the detection conditions of the four approaches (ROR: lower limit of 95% CI > 1, PRR: *χ*2 ≧4, lower limit of 95% CI > 1, EBGM. EBGM05 > 2, BCPNN: IC025 > 0) were regarded as potential AE signals of GLP-1RAs. Formulas for disproportionate analysis shown in Supplementary Table S1. All data processing and statistical analysis performed using R (version 4.23), Microsoft Excel (version 2021).

### 2.3 Statistical methods

The study compared whether there were significant differences in proportion of gender, age, and weight across AEs between the serious and non-serious outcomes among the different medications (including albiglutide, exenatide, dulaglutide, liraglutide, semaglutide, and tirzepatide). Proportions were compared using Pearson’s chi-squared (χ2) or Fisher’s exact test, and the Mann-Whitney *U* test was applied for continuous non-normal distribution data, such as age and weight. Time to onset (TTO) was defined as the interval between the date of AE onset (EVENT_DT in the DEMO file) and the date of the start of drug use (START_DT in the THER file). Once data containing input errors (for example, where the EVENT_DT was earlier than the START_DT), inaccurate date inputs or missing specific data were excluded. Statistical analyses were performed to obtain the median time to onset of the target AEs, quartiles, and parameters of the Weibull test. The test results were analyzed with reference to previous studies in order to obtain the pattern of AE occurrence (Kinoshita et al., 2020; Mazhar et al., 2021). Data were analyzed using SPSS (version 22.0), and Minitab (version 21), and statistical significance was set at *p* < 0.05.

## 3 Results

### 3.1 Descriptive analysis

The AEs for metabolic and nutritional disorders associated with albiglutide (n = 240), exenatide (n = 10,450), dulaglutide (n = 4,847), liraglutide (n = 2,860), semaglutide (n = 2,905), tirzepatide (n = 1,089), and lixisenatide (n = 13), were screened according to their date of launch. Different GLP-1 RAs AE characteristics are summarized in Table 1. More metabolic and nutritional disease adverse reaction reports occurred in female (n = 13,817, 61.67%) compared to male patients (n = 7,684, 34.30%). More patients within a weight distribution of >100 kg (n = 3,523, 15.72%) with a median weight of 95 (IQR 81.6–112) kg. The age distribution of more patients was 45–65 years (n = 7,880, 35.17%), with a median age of 61 (IQR 53–68) years. The vast majority of the AEs were reported in patients from the United States (n = 19,807, 88.41%), and reports of AEs were submitted primarily by consumers (n = 17,775, 79.34%), followed by physicians (n = 2,328, 10.39%). Hospitalization (n = 3,961, 48.37%) and other serious (important medical events) (n = 3,395, 41.46%) were the most frequently reported serious outcomes.

**TABLE 1:** Reported characteristics of adverse events in metabolism and nutrition associated with GLP-1RAs.

Characteristics	All GLP-1Ras (n = 22,404)	Exenatide (n = 10,450)	Liraglutide (n = 2,860)	Albiglutide (n = 240)	Dulaglutide (n = 4,847)	Semaglutide (n = 2,905)	Tirzepatide (n = 1,089)	Lixisenatide (n = 13)
Gender, n (%)								
Female	13,817 (61.67)	6,691 (64.03)	1,938 (67.76)	147 (61.25)	2,598 (53.60)	1,750 (60.24)	689 (63.27)	4 (30.77)
Male	7,684 (34.30)	3,654 (34.97)	883 (30.87)	85 (35.42)	1,753 (36.17)	1,072 (36.90)	228 (20.94)	9 (69.23)
Unknown	903 (4.03)	105 (1.00)	39 (1.36)	8 (3.33)	496 (10.23)	83 (2.86)	172 (15.79)	0 (0.00)
Weight (kg), n (%)								
<80	2,025 (9.04)	1,449 (7.95)	219 (7.66)	9 (3.75)	152 (3.14)	183 (6.30)	10 (0.92)	3 (23.08)
80–100	3,235 (14.44)	2,618 (13.63)	232 (8.11)	10 (4.17)	138 (2.85)	209 (7.19)	27 (2.48)	1 (7.69)
>100	3,523 (15.72)	2,993 (45.98)	223 (7.80)	11 (4.58)	117 (2.41)	168 (5.78)	11 (1.01)	0 (0.00)
Unknown	13,621 (60.80)	3,390 (32.44)	2,186 (76.43)	210 (87.5)	4,440 (91.60)	2,345 (80.72)	1,041 (95.60)	9 (69.23)
Median (kg)	95 (81.6–112)	96.12 (82.55–113.38)	89.9 (74.6–107.5)	89.63 (77.69–114.97)	87.7 (71–102.4)	88.95 (74.8–104.98)	103.75 (85.15–130.08)	63 (55.257–8.25)
Age (years), n (%)								
<18	11 (0.05)	2 (0.01)	7 (0.24)	0 (0.00)	2 (0.04)	0 (0.00)	0 (0.00)	0 (0.00)
18–44	1,301 (5.81)	509 (4.87)	263 (9.20)	11 (4.58)	215 (4.44)	203 (6.99)	100 (9.18)	0 (0.00)
45–65	7,880 (35.17)	4,683 (44.81)	1,080 (37.76)	90 (37.50)	976 (20.14)	814 (28.02)	233 (21.40)	4 (30.77)
>65	4,718 (21.06)	2,351 (22.50)	632 (22.10)	38 (15.83)	838 (17.30)	789 (27.16)	64 (5.88)	6 (46.15)
Unknown	8,494 (37.91)	2,905 (27.80)	878 (30.70)	101 (42.08)	2,816 (58.10)	1,099 (37.83)	692 (63.54)	3 (23.08)
Median (years)	61 (53–68)	61 (54–67)	60 (51–68)	60 (53–66)	63 (54–71)	63 (54–72)	53 (44–62)	67 (50.25–71.25)
Indications, n (%)								
Type 2 diabetes mellitus	4,431 (19.78)	1,780 (17.03)	904 (32.87)	152 (63.33)	351 (7.24)	968 (33.32)	271 (24.89)	5 (38.46)
Diabetes mellitus	928 (4.12)	391 (3.74)	133 (4.65)	36 (15.00)	66 (1.36)	230 (7.92)	67 (6.15)	5 (38.46)
Others	4,778 (21.33)	289 (2.77)	2,113 (73.88)	50 (20.83)	145 (2.99)	1,431 (49.26)	747 (68.60)	3 (23.08)
Unknown	15,338 (68.46)	10,161 (97.23)	610 (2.13)	2 (0.83)	4,285 (88.41)	276 (9.50)	4 (0.37)	0 (0.00)
Occupation of reporters, n (%)								
Consumer	17,775 (79.34)	8,241 (78.86)	2,008 (70.21)	216 (90.00)	4,287 (88.45)	2,024 (69.67)	995 (91.37)	4 (30.77)
Physician	2,328 (10.39)	984 (9.42)	485 (16.96)	16 (6.67)	292 (6.02)	498 (17.14)	46 (4.22)	7 (53.85)
Health professional	416 (1.86)	16 (0.15)	61 (2.13)	0 (0.00)	115 (2.37)	196 (6.75)	27 (2.48)	1 (7.69)
Pharmacist	458 (2.04)	122 (1.17)	89 (3.11)	1 (0.42)	88 (1.82)	147 (5.06)	10 (0.92)	1 (7.69)
Other health-professional	506 (2.26)	227 (2.17)	189 (6.61)	2 (0.83)	51 (1.05)	37 (1.27)	0 (0.00)	0 (0.00)
Lawyer	11 (0.05)	7 (0.07)	2 (0.07)	0 (0.00)	1 (0.02)	1 (0.03)	0 (0.00)	0 (0.00)
Unknown	900 (4.02)	853 (8.16)	26 (0.91)	5 (2.08)	13 (0.27)	2 (0.07)	1 (0.09)	0 (0.00)
Reported countries, n (%)								
US	19,807 (88.41)	9,806 (93.84)	2,285 (79.90)	229 (95.42)	4,034 (83.23)	2,392 (82.34)	1,061 (97.43)	0 (0.00)
Non-US	2,581 (11.52)	631 (6.04)	575 (20.10)	11 (4.58)	813 (16.77)	513 (17.66)	28 (2.57)	13 (100.00)
Unknown	16 (0.07)	13 (0.12)	0 (0.00)	0 (0.00)	0 (0.00)	0 (0.00)	0 (0.00)	0 (0.00)
Outcomes, n (%)								
Non-serious outcome	15,849 (65.93)	8,229 (73.98)	1,738 (55.33)	158 (58.52)	3,124 (60.13)	1,689 (52.93)	911 (81.56)	0 (0.00)
Serious outcome	8,189 (34.07)	2,895 (26.02)	1,403 (44.67)	112 (41.48)	2,071 (39.87)	1,502 (47.07)	206 (18.44)	0 (0.00)
Death	249 (3.04)	87 (3.01)	48 (3.42)	1 (8.93)	82 (3.96)	25 (1.66)	6 (2.91)	0 (0.00)
Disability	171 (2.09)	42 (1.45)	30 (2.14)	2 (1.79)	46 (2.22)	49 (3.26)	2 (0.97)	0 (0.00)
Hospitalization	3,961 (48.37)	1,466 (50.64)	674 (48.04)	32 (28.57)	992 (47.90)	686 (45.67)	105 (50.97)	6 (37.50)
Life-Threatening	374 (4.57)	124 (4.28)	85 (6.06)	7 (6.25)	94 (4.54)	55 (3.66)	9 (4.37)	0 (0.00)
Other serious (Importent medical event)	3,395 (41.46)	1,154 (39.86)	562 (40.06)	70 (62.50)	853 (41.19)	664 (44.21)	82 (39.81)	10 (62.50)
Required intervention to prevent permanent impairment/damage	52 (0.63)	22 (0.76)	1 (0.07)	0 (0.00)	4 (0.19)	23 (1.53)	2 (0.97)	0 (0.00)
Congenital anomaly	3 (0.04)	0 (0.00)	3 (0.21)	0 (0.00)	0 (0.00)	0 (0.00)	0 (0.00)	0 (0.00)

N, number of total gastrointestinal adverse event reports.

### 3.2 Different GLP-1 RA-related signals

Three GLP-1 RAs displayed positive SOC signals in Table 2: semaglutide (ROR, 3.34; 95%CI, 3.22), liraglutide (ROR, 2.78; 95%CI, 2.69), and exenatide (ROR, 2.15; 95%CI, 2.11) showed an association with metabolism and nutrition AEs. In contrast, lixisenatide (ROR, 2.85; 95% CI, 1.66), tirzepatide (ROR, 2.11; 95% CI, 1.99), dulaglutide (ROR, 2.04; 95% CI, 1.99) and albiglutide (ROR, 0.60; 95% CI, 0.53), did not show any association.

**TABLE 2:** Signal detection for GLP-1 RAs-associated metabolic and nutritional adverse events.

GLP-1 RA	The report number	ROR (95% CI)	PRR (95% CI)	EBGM (EBGM05)	IC (IC025)
Albiglutide	264	0.60 (0.53)	0.61 (0.54)	0.61 (0.55)	−1.39 (−0.90)
Exenatide	11,631	2.15 (2.11)	2.10 (2.06)	2.09 (2.06)	0.94 (1.04)
Liraglutide	3,222	2.78 (2.69)	2.68 (2.60)	2.68 (2.60)	0.70 (1.37)
Dulaglutide	5,418	2.04 (1.99)	2.00 (1.95)	1.99 (1.95)	1.01 (0.95)
Lixisenatide	14	2.85 (1.66)	2.75 (1.65)	2.75 (1.75)	0.69 (0.53)
Semaglutide	3,305	3.34 (3.22)	3.19 (3.09)	3.18 (3.09)	0.60 (1.62)
Tirzepatide	1,189	2.11 (1.99)	2.07 (1.95)	2.06 (1.96)	0.96 (0.95)

PRR, the proportional reporting ratio; ROR, the reporting odds ratio; IC, the information component; EBGM, the empirical Bayes geometric mean; CI, confidence interval; 95% CI, two-sided for ROR, c2, chi-squared; IC025 and EBGM05 lower one-sided for IC, and EBGM.

Decreased appetite was the most frequently reported AE in the FAERS database, including exenatide (n = 6,125, 58.61%), dulaglutide (n = 2,331, 48.09%), liraglutide (n = 1,174, 41.05%), semaglutide (n = 1,377, 47.40%), and tirzepatide (n = 550, 50.51%), with the exception that it is also one of the therapeutic effects of GLP-1 RAs, therefore, decreased appetite was analyzed only at the HLT level (Supplementary Table S2).

**FIGURE 2:**
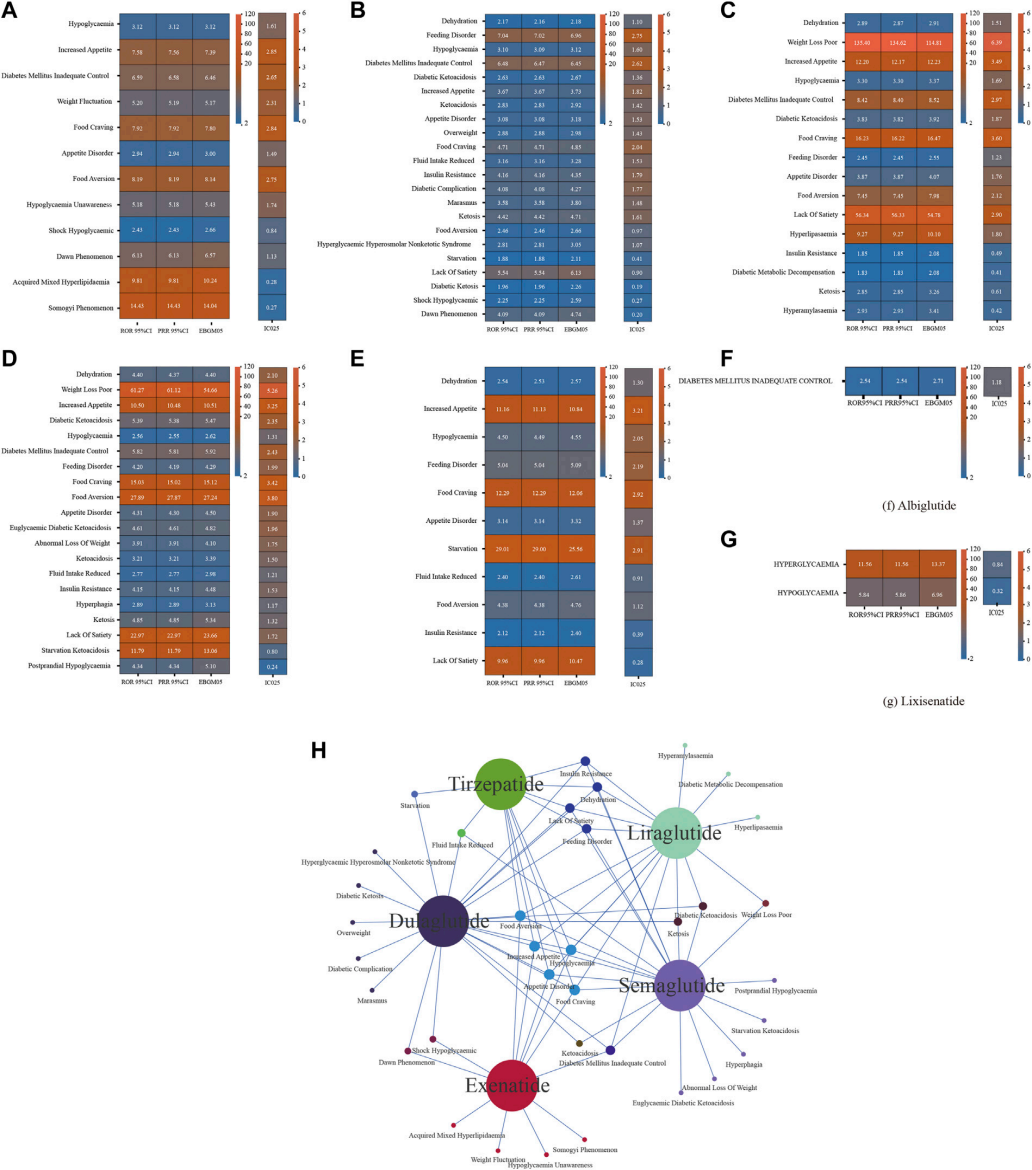
Results of disproportionate analysis of AEs associated with metabolism and nutrition of seven GLP-1 RAs at the PT level. **(A)** ROR 95%CI, PRR 95%CI, EBGM05, IC025 for exenatide metabolism and nutrition-associated AEs. **(B)** ROR 95%CI, PRR 95%CI, EBGM05, IC025 for liraglutide metabolism and nutrition-associated AEs. **(C)** ROR 95%CI, PRR 95%CI, EBGM05, IC025 for dulaglutide metabolism and nutrition-associated AEs. **(D)** ROR 95%CI, PRR 95%CI, EBGM05, IC025 for semaglutide metabolism and nutrition-associated AEs. **(E)** ROR 95%CI, PRR 95%CI, EBGM05, IC025 for tirzepatide metabolism and nutrition-associated AEs. **(F)** ROR 95%CI, PRR 95%CI, EBGM05, IC025 for albiglutide metabolism and nutrition-associated AEs. **(G)** ROR 95%CI, PRR 95%CI, EBGM05, IC025 for lixisenatide metabolism and nutrition-associated AEs. **(H)** Overlap relationship between the positive signals of five GLP-1RAs at the pt levels. GLP-1RA, glucagon-like peptide-1 receptor agonist; AE, adverse event; PT, preferred term; ROR, reporting odds ratio; PRR, proportional reporting rate; EBGM05, lower one-sided 95% confidence limit (95% CI) of the empirical Bayes geometric mean; IC025, lower bound of the information component for the Bayesian confidence propagation neural network.

We disproportionately analyzed each GLP-1RA separately at the PT level and identified positive signals according to the screening criteria (Figure 2). Albiglutide (n = 240) and lixisenatide (n = 9) were associated with low numbers of metabolic and nutritional AEs, with one and two signals detected, respectively. In contrast, exenatide, dulaglutide, liraglutide, semaglutide, and tirzepatide were associated with 12, 22, 16, 20, and 11 signals, respectively; Including episodes of food craving, hypoglycaemia, appetite disorder, increased appetite, and food aversion for all five drugs. Notably, IC025 > 3 was detected for eight PTs by the BCPNN approach: liraglutide: weight loss poor, food craving, increased appetite; semaglutide: weight loss poor, food aversion, food craving, increased appetite; tirzepatide: increased appetite.

### 3.3 Severe *versus* non-severe cases

As shown in Table 3, with the exception of lixisenatide (n = 14), which was not included because the number of AEs was insufficient for analysis, differential analyses of gender, age, and body weight were performed between severe and non-severe cases in patients with AEs of metabolism and nutrition disorders who were treated with the remaining six GLP-1 RAs. The results showed statistically significant differences between two groups, included gender (χ^2^ = 13.776, *p* < 0.001), age (60 vs 62 years; *p* = 0.011), and body weight (94.43 vs 96.6 kg, *p* = 0.016) for exenatide; gender (χ^2^ = 25.577, *p* < 0.001) for liraglutide; gender (χ^2^ = 37.710, *p* < 0.001) and body weight (85 vs 96.15 kg, *p* = 0.003) for dulaglutide; age (61 vs 65 years; *p* < 0.001) and body weight (88 vs 93 kg, *p* = 0.018) for semaglutide; gender (χ^2^ = 11.664, *p* < 0.001) for tirzepatide.

**TABLE 3:** Differences in clinical characteristics of serious and non-serious reports.

Drug		Serious cases	Non-serious cases	Statistic	*p* -value
Exenatide					
	Gender, n (%)				
	Male	852 (23.32)	2,802 (76.68)	13.776^b^	<0.001^a^
	Female	1,351 (20.19)	5,340 (79.81)		
	Age, years (median, IQR)	60 (52–68)	62 (54–69)	−2.534^d^	0.011^c^
	Weight, kg (median, IQR)	94.43 (81.63–112.49)	96.6 (83.01–113.40)	−2.415^d^	0.016^c^
**Liraglutide**					
	Gender, n (%)				
	Male	407 (46.09)	476 (53.91)	25.577^b^	<0.001^a^
	Female	699 (36.07)	1,239 (63.93)		
	Age, years (median, IQR)	60 (50–68)	60 (52–68)	−0.565^d^	0.572^c^
	Weight, kg (median, IQR)	89 (73–106.6)	91.2 (79.6–112.45)	−1.773^d^	0.076^c^
**Albiglutide**					
	Gender, n (%)				
	Male	32 (37.65)	53 (62.35)	0.595^b^	0.441^a^
	Female	48 (32.65)	99 (67.35)		
	Age, years (median, IQR)	61.5 (53.25–69)	59 (53–65)	−1.465^d^	0.143^c^
	Weight, kg (median, IQR)	89.63 (78.47–116.66)	88.67 (73.47–109.34)	−0.674^d^	0.527^c^
**Dulaglutide**					
	Gender, n (%)				
	Male	745 (42.50)	1,008 (57.50)	37.710^b^	<0.001^a^
	Female	866 (33.33)	1732 (66.67)		
	Age, years (median, IQR)	62 (51.75–72)	63 (55–71)	−1.237^d^	0.216^c^
	Weight, kg (median, IQR)	85.00 (70.39–101.11)	96.15 (77.10–115.47)	−2.940^d^	0.003^c^
**Semaglutide**					
	Gender, n (%)				
	Male	471 (43.94)	601 (56.06)	2.587^b^	0.108^a^
	Female	715 (40.86)	1,035 (59.14)		
	Age, years (median, IQR)	61 (51–71)	65 (57–72)	−5.475^d^	<0.001^c^
	Weight, kg (median, IQR)	88 (74–103)	93 (77.6–111)	−2.357^d^	0.018^c^
**Tirzepatide**					
	Gender, n (%)				
	Male	55 (24.12)	173 (75.88)	11.664^b^	<0.001^a^
	Female	99 (14.37)	590 (85.63)		
	Age, years (median, IQR)	53 (44–61)	53 (44–62)	−0.505^d^	0.614^c^
	Weight, kg (median, IQR)	103.75 (85.5–120.25)	107.8 (69.75–135.88)	−0.454^d^	0.65^c^

^a^ Proportions were compared using Pearson χ2 test.

^b^ The χ2 statistic of the Pearson chi-square test.

^c^ Mann-Whitney *U* test.

^d^ The Z statistic of the Mann-Whitney *U* test.

*P* -value <0.05 were considered statistically significant.

A more detailed summary of the results in Supplementary Table S3 indicate that 1, 7, 11, 15, 13, and 3 AEs of the six GLP-1 RAs (albiglutide, exenatide, liraglutide, dulaglutide, semaglutide, and tirzepatide) tended to be reported as severe AEs (*p* < 0.05), respectively. Dehydration was the AE with the highest number of reports for severe outcomes of GLP-1RAs, including liraglutide (n = 318, 23.93%), dulaglutide (n = 434, 20.90%), semaglutide (n = 370, 25.10%), tirzepatide (n = 70, 32.86%). Of note, all AE outcomes were severe, including dulaglutide: ketoacidosis (n = 58), marasmus (n = 17), hyperglycaemic hyperosmolar nonketotic syndrome (n = 12), diabetic ketosis (n = 4); liraglutide: diabetic ketoacidosis (n = 104), hyperlipasaemia (n = 9), diabetic metabolic decompensation (n = 6), hyperamylasamia (n = 4); albiglutide: diabetic mellitus inadequate control (n = 23); semaglutide: euglycemic diabetic ketoacidosis (n = 32), ketoacidosis (n = 28), and ketosis (n = 8), starvation ketoacidosis (n = 4).

### 3.4 Time to onset analysis

**FIGURE 3:**
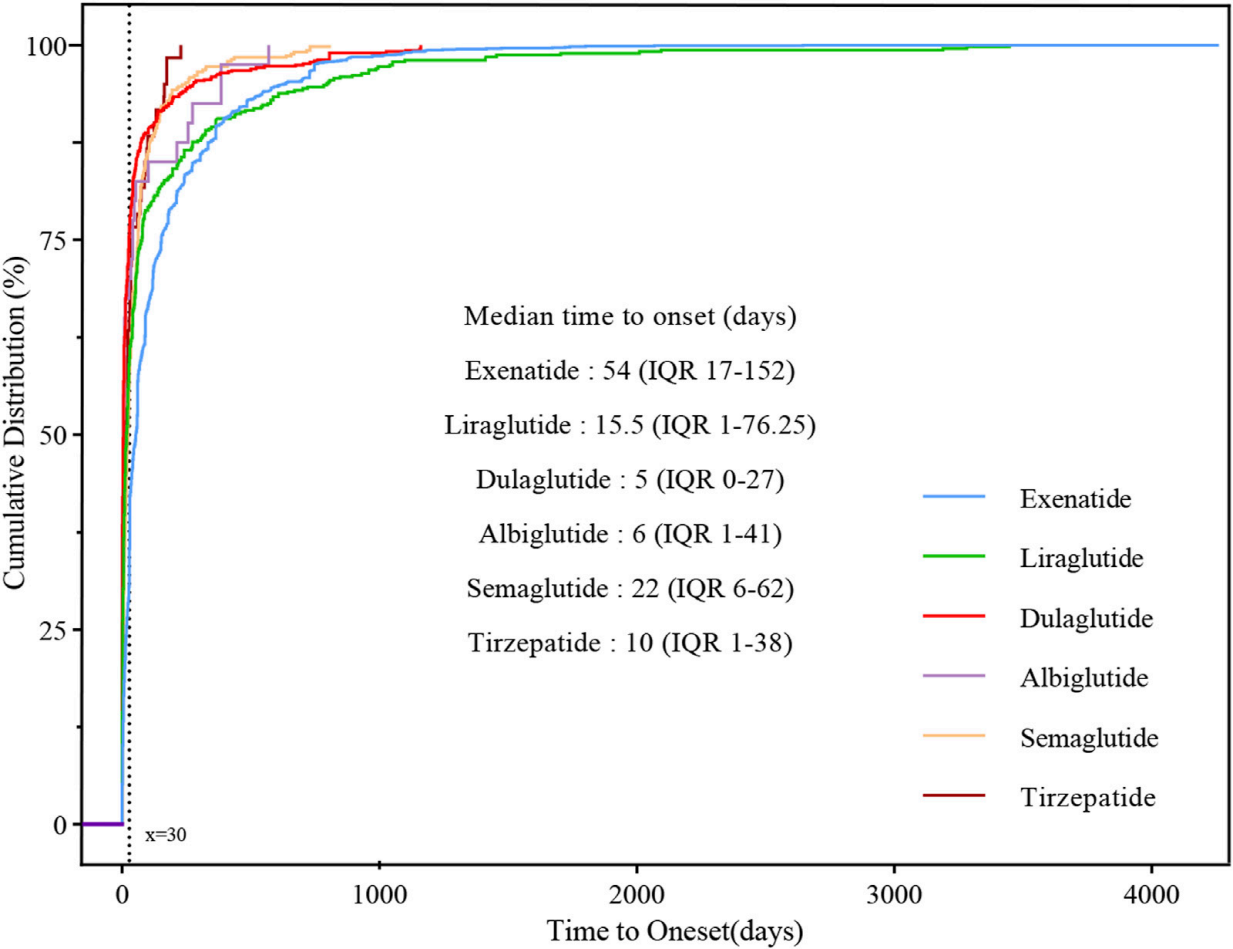
Cumulative distribution curves demonstrating the onset time of glucagon-like peptide-1 receptor agonist (GLP-1 RA)-related metabolic and nutritional adverse events after treatment with different GLP-1 RAs.

With the exception of lixisenatide, which was excluded from the analysis due to insufficient validated data, the time to onset of metabolic and nutritional disorders was summarized in Figure 3. Median time to onset for exenatide was the longest at 54 (IQR: 17–152) days, and dulaglutide was the shortest at 5 (IQR: 0–27) days, 15.5 (IQR: 1–76.25) days for liraglutide, 6 (IQR: 1–41) days for albiglutide, 22 (IQR: 6–62) days for semaglutide and 10 (IQR: 1–38) days for tirzepatide. Cumulative distribution curves indicate the time to onset of metabolic and nutritional AEs after treatment with different GLP-1 RAs, and the difference was significant in the exenatide group compared to the other five GLP-1 RAs (*p* < 0.001).

Given that dehydration was the AE in which the most severe outcomes occurred after the use of multiple GLP-1 RAs, we performed an analysis of three GLP-1 RAs with sufficient data on time to onset in the occurrence of dehydration for analysis (Table 4), and the median time to onset was, in descending order, dulaglutide (5 days, IQR 0.5–38.5), liraglutide (20 days, IQR 4.25–64.75), and semaglutide (26 days, IQR 6–61). In the assessment of Weibull 3-parameter analysis, all shape parameters β and its upper limit of 95% CI were <1.

**TABLE 4:** Time to onset analysis of dehydration after treatment with three GLP-1RAs.

Drug		Weibull distribution						Failure type
	Cases	TTO (days)		Shape parameter		Scale parameter		
	N	Median (IQR)	Min–max	Β	95% CI	Α	95% CI	
Liraglutide	96	20 (4.25–64.75)	0–994	0.46	0.39–0.53	35.38	22.35–56.01	Early failure
Dulaglutide	93	5 (0.5–38.5)	0–1,161	0.17	0.14–0.21	4.61	1.36–15.55	Early failure
Semaglutide	85	26 (6–61)	0–716	0.58	0.49–0.69	33	22.51–48.38	Early failure

n, number of cases with available time-to-onset; IQR, interquartile range; TTO, Time-to-onset. When TTO, is 0 days, the adverse event occurred within the same day with the therapy.

## 4 Discussion

We analyzed data on post-marketing AEs reported in the FAERS database related to the use of GLP-1 RA. Specifically, we adopted disproportionality analysis to identify AEs highly associated with GLP-1 RA post-treatment. We focused on AEs with severe outcomes, while also analyzing the time of onset of the six GLP-1 RAs. A Weibull 3-parameter analysis was employed to examine reports of severe outcomes associated with dehydration to identify temporal patterns of time to onset. Our study summarized the metabolic and nutritional class of AEs associated with GLP-1 RAs.

### 4.1 Signals of adverse events

We observed that metabolic and nutritional adverse reactions were more frequently reported by females (Table 1), which has previously been interpreted as a greater inclination for females to report AEs to FAERS (Becker, 2022; Chen et al., 2024). Semaglutide (ROR = 3.34) had a high risk of inducing metabolic and nutritional AEs and the most pronounced risk of gastrointestinal adverse effects, which may be related to the fact that the SOC tier of semaglutide had the highest disproportionate signal intensity (Liu et al., 2022).

Exenatide (n = 10,450), dulaglutide (n = 4,847), liraglutide (n = 2,860), semaglutide (n = 2,905), and tirzepatide (n = 1,089) had the greatest number of reported AEs, and the highest number of AEs reported on the effects of appetite, as reflected in our analyses of the HLT stratum (Supplementary Table S2). This is consistent with the fact that this class of drugs produces significant appetite suppression in response to central and peripheral GLP-1 R activation and has been actively used in the treatment of T2DM, obesity, and overweight (Shah and Vella, 2014; Coskun et al., 2018; Brunton and Wysham, 2020; Aldawsari et al., 2023; Bailey et al., 2023). Decreased appetite is common in GLP-1 RAs use and has been demonstrated not to be driven by gastric emptying and gastrointestinal adverse effects resulting from treatment with short- or long-acting GLP-1 RAs (Quast et al., 2021). Unfortunately, anorexia produced by the agitation of the central system GLP-1 receptor is undesired in lean T2DM patients (Borner et al., 2022). Disproportionate signals like increased appetite, food craving, hyperphagia, lack of satiety, and appetite disorder were also detected. Appetite disorder was mined in pharmacovigilance of adverse psychiatric events with GLP-1 RA (Chen et al., 2024), we hypothesized that other effects, such as increased appetite and anorexia, could have arisen during agonism of GLP-1 receptor in the nucleus of the solitary tract (NTS). In addition, different weight loss interventions have different effects on appetite, with low-calorie diets and exercise causing an increase in appetite, and medication (GLP-1 RAs) and bariatric surgery causing decreased appetite (Papamargaritis et al., 2024). Consequently, it is necessary to evaluate the positive and negative effects of GLP-1 RAs on appetite in different patients before using them.

Our study identified a potential risk of dehydration with dulaglutide, liraglutide, semaglutide, and tirzepatide, semaglutide emerged as having the highest risk of various fluid reductions (Supplementary Table S2). In animal experiments, it has been demonstrated that the endogenous GLP-1 receptor system is involved in the control of fluid intake and that, unlike food-generated stimuli, both endogenous GLP-1 and exogenous GLP-1 Ras inhibit fluid intake by activating the central nervous system GLP-1 receptor (McKay et al., 2011; McKay et al., 2014), all of which evidence suggests that GLP-1 RAs are associated with dehydration (Filippatos et al., 2014), which is consistent with the results of the two subsequent clinical trials (Winzeler et al., 2020; Winzeler et al., 2021). Meanwhile, the most frequently reported adverse effects of GLP-1 RAs are gastrointestinal, including nausea, vomiting, and diarrhea (Liu et al., 2022; Chong et al., 2024), all of which predispose patients to dehydration. However, current studies of fluid intake inhibition for GLP-1 RAs are based on routinely administered doses, and little is known about indications such as obesity and overweight for taking higher doses.

In previous real-world studies of GLP-1 RAs-induced hypoglycemia, lixisenatide showed the strongest association (Zhao et al., 2021), consistent with our finding (Figure 2). Not only that, but the risk of hyperglycaemic conditions and hypoglycaemic conditions with lixisenatide was also the highest in our study (Supplementary Table S2). Although GLP-1 RAs by themselves have a low risk of hypoglycemia, when used in combination with sulfonylureas and/or insulin, some experts recommend lowering the dose of sulfonylureas and short-acting and low-acting pancreatic islet analogs before or during the use of GLP-1 RAs (Romera et al., 2019; Smits and Van Raalte, 2021).

Corresponding to the signal intensity for weight loss poor of GLP-1 RAs in our study, a network meta-analysis showed a decreasing trend in weight loss efficacy for tirzepatide, semaglutide, and liraglutide (Pan et al., 2024). The weight loss effect produced by liraglutide in previous studies was individualized across subjects, with more significant fat mass reductions in females, and the starting point for measurement of the weight loss effect is recommended to start from baseline (Santini et al., 2023; Schultes et al., 2024). Differences in individual specificity and tolerance to GLP-1 RAs may be the main reason for differences in weight loss (Ruder, 2023), with racial differences also contributing (Dobbie et al., 2024). Meanwhile, the recent observation that previous use of anti-obesity medication is associated with poorer weight loss outcomes with semaglutide further expands our knowledge that GLP-1 RAs produce poor weight loss (Ghusn et al., 2024).

### 4.2 Severe versus non-severe cases

The most frequently reported adverse effects associated with GLP-1 RAs were gastrointestinal in nature. Practice guides suggest that these effects can be minimized by gradual dose escalation and effective prevention or mitigation during initiation of treatment, generally resulting in mild or moderate adverse events (Brunton and Wysham, 2020). In recent years, the incidence of cases associated with GLP-1 RAs has increased, as well as the rates of serious medical outcomes and healthcare facility admissions, especially for semaglutide and liraglutide (Gaw et al., 2024). In our study involving both severe and non-severe cases, the analysis of patient age and weight suggested a higher risk of severe metabolic and nutritional AEs in patients with younger age and lighter weight. Our study also shows that GLP-1 RA-associated metabolic and nutritional adverse events appear to be defined worse for women. We failed to find relevant studies to corroborate our findings on weight, age or gender, requiring clinical studies to validate our findings.

In the analysis of serious *versus* non-serious outcomes in cases, 1, 7, 11, 15, 13, and 3 AEs tended to be reported as serious AEs for albiglutide, exenatide, liraglutide, dulaglutide, semaglutide, and tirzepatide, respectively (Supplementary Table S3). Unexpectedly, dehydration produces the highest number of cases with serious outcomes in liraglutide, dulaglutide, semaglutide, and tirzepatide. It is apparent that the inhibition of fluid intake in the CNS produced by the administration of GLP-1 RAs and the loss of fluid induced by gastrointestinal adverse effects combine to contribute to the onset of dehydration. In elderly patients, who are at inherently higher risk of developing diabetes and dehydration (Atciyurt et al., 2024), low fluid intake and hypovolemia are the main etiologic factors contributing to severe dehydration outcomes in elderly patients (Lambert and Carey, 2023). Reduced fluid intake and gastrointestinal adverse effects due to GLP-1 RAs are potential triggers of acute kidney injury (Filippatos et al., 2014; Long et al., 2024), all of which contribute to the significant number of severe outcomes of dehydration following GLP-1 RA use. At the same time, there was no positive signal for dehydration and fluid intake inhibition with exenatide, suggesting that exenatide is the better drug option in patients at risk for dehydration.

In our study, AEs submitted by multiple GLP-1 RAs regarding ketoacidosis were disproportionate and nearly all AEs had a severe outcome when the effect of the combination was not considered (Supplementary Table S3). In a case report of ketoacidosis resulting from an injection of Wegovy 1.7 mg for the treatment of type 1 diabetes mellitus (T1DM), the effects of appetite suppression and inhibition of gluconeogenesis and glycogenolysis produced by GLP-1 RAs contributed to the reduction of the patient’s insulin push and induced the development of diabetic keto acidosis. Moreover, this could become more prevalent with the widespread use of GLP-1 RAs (Widhalm and Pulido, 2023). A meta-analysis of GLP-1 RAs as adjuvant therapy in patients with T1DM also observed that liraglutide was associated with higher odds of ketosis (OR 1.8; 95% CI, 1.1–2.8) (Park et al., 2023). Coupled with the slightly disproportionate reporting of diabetic ketoacidosis associated with GLP-1 RAs when they occur without combination with insulin (Yang et al., 2022), there is a need to be concerned about ketoacidosis as a potential adverse effect when using GLP-1 RAs.

### 4.3 Time to onset analysis

In TTO analysis, the median time to onset of GLP-1 RAs except exenatide (54 days, IQR 17–152), liraglutide (15.5 days, IQR 1–76.25), dulaglutide (5 days, IQR 0–27), albiglutide (6 days, IQR 1–41), semaglutide (22 days, IQR 6–62) and tirzepatide (10 days, IQR 1–38) all within 30 days, and the difference in onset of action between exenatide and other GLP-1 RAs was statistically significant (*p* < 0.001). The pharmacokinetic and pharmacodynamic characteristics of different GLP-1 RAs may be relevant (Chen et al., 2024), no studies have characterized the time to onset of metabolic and nutritional adverse effects of GLP-1 RAs, and we look forward to more clinical practice to validate our results in the future. The Weibull parameter is used to predict the time to onset and can serve to refine pharmacologic management thinking for patients in clinical practice (Mazhar et al., 2021; Shu et al., 2022). The shortest median time to onset of dehydrated AEs was for dulaglutide (5 days, IQR 0.5–38.5), followed by liraglutide (20 days, IQR 4.25–64.75) and semaglutide (26 days, IQR 6–61). Dehydration with three GLP-1 RAs showed early failure-type characteristics, suggesting that the risk of developing dehydration decreased progressively over time.

The desire to explore the effects associated with appetite and feeding prompted further differential analyses of whether metabolic and nutritional AEs are among the AEs with appetite disorders (Supplementary Table S4), which revealed that women appeared to be more susceptible to appetite-related side effects with the use of GLP-1 RAs. On the one hand, estradiol in women can mediate a reduction in food intake through multiple pathways (Eckel, 2011), an activational effect of estrogens may cause anorexia nervosa (Asarian and Geary, 2013). On the other hand, the literature reports a much greater prevalence of psychogenic eating disorders in women than in men in the United States, including anorexia nervosa, bulimia nervosa, and binge eating disorder (Hudson et al., 2007; Guerdjikova et al., 2019). Given the remarkable efficacy of GLP-1 RAs in the treatment of binge eating in several trials, although no GLP-1 RA has been approved for the treatment of any eating disorder symptom, GLP-1 RAs remain a highly promising drug for the treatment of bulimia at this time (Bartel et al., 2024). Based on other potential adverse effects of GLP-1 RAs and positive and/or negative effects on eating disorders (Richards and Khalsa, 2024), we suggest that careful assessment of a patient’s appetite status is necessary when considering GLP-1 RAs for the treatment of eating disorders, especially in female patients.

### 4.4 Limitations

In terms of the data received in the FAERS database, it is a spontaneous reporting system, and involving reports submitted by different reporters introduce the potential for duplicate records and variable quality of information. Despite our effect on data corrections and deletions of duplication, the large number of missing data services link the quality of data and its subsequent findings to varying degrees. Some of the detected disproportionate signals of adverse reactions overlapped somewhat with the clinical manifestations of the therapeutic drug indications, which prevented our study from distinguishing between them. We selected post-market real-world data of individual GLP-1 RAs for analysis, although this approach provides a more accurate description of the characteristics of each GLP-1 RA, it does not allow us to evaluate the overall intensity and magnitude of targeting a specific drug with disproportionate signaling effects. Finally, the FAERS-based disproportionality analysis reflects only an assessment of signal intensity, which is a statistical association that needs to be validated by further studies (Liu et al., 2022).

## 5 Conclusion

In this study, we quantitatively analyzed the relationship between each GLP-1 RA and metabolic and nutritional AEs based on the FAERS database at multiple levels and from various perspectives. Exenatide, liraglutide, and semaglutide were associated with metabolic and nutritional AEs, and multiple GLP-1 RAs novel AE signals were identified by disproportionate analysis. TTO analysis suggests metabolic and nutritional AEs of exenatide tend to develop later than other GLP-1 RAs. The occurrence of dehydration following the use of dulaglutide, liraglutide, and semaglutide tends to be within a month. However, the serious outcomes associated with the development of dehydration following the use of GLP-1 RAs warrant increased attention.

## Data Availability

Publicly available datasets were analyzed in this study. This data can be found here: https://fis.fda.gov/extensions/FPD-QDE-FAERS/FPD-QDE-FAERS.html.

## Abbreviations

AEs, Adverse Events; BCPNN, Bayesian Confidence Propagation Neural Network; EBGM, Empirical Bayesian Geometric Mean; EMA, European Medicines Agency; FAERS, FDA Adverse Event Reporting System; FDA, United States Food and Drug Administration; GLP-1 RAs, Glucagon-like peptide-1 receptor agonists; HLT, High level term; IDF, The International Diabetes Federation; IQR, Interquartile Range; MedDRA, Medical Dictionary for Regulatory Activities; NTS, Nucleus of the solitary tract; PRR, Proportional reporting ratios; PT, Preferred term; ROR, Reporting ratio of ratios; SOC, System organ class; SSIBs, Suicidal or self-injurious behaviors; T1DM, Type 1 diabetes mellitus; T2DM, Type 2 diabetes mellitus; TTO, Time to onset.
