# Supplementary material for: Pharmacovigilance study of GLP-1 receptor agonists for metabolic and nutritional adverse events
Source: Front Pharmacol. 2024 Jul 8;15:1416985. doi: 10.3389/fphar.2024.1416985 (PMC11260617; doi:10.3389/fphar.2024.1416985)
Supplement: Supplementary file 1 [file Table1.DOCX]

Supplementary Material

Pharmacovigilance Study of GLP-1 Receptor Agonists for Metabolic and Nutritional Adverse Events

**Long He*****, Qiuyu Li*****, Yongfeng Yang, Jiahao Li, Wei Luo, Yilan Huang, Xiaoyan Zhong**

*** Correspondence:** Yilan Huang: [hyl3160131@126.com](mailto:hyl3160131@126.com), Xiaoyan Zhong: [312556662@qq.com](mailto:312556662@qq.com)

# Supplementary Data

Supplementary Table 1. Four methods of disproportionality analysis.

| **Algorithms** | **Equation** | **Criteria** |
| --- | --- | --- |
| **ROR** | 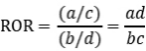  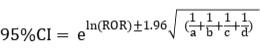 | a≥3, lower limit of 95%Cl＞1 |
| **PRR** | 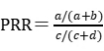  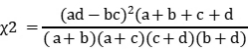 | a≥3, PRR≥2 |
| **BCPNN** | IC=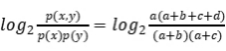  E(IC)=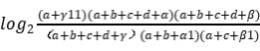  V(IC)=  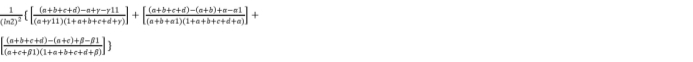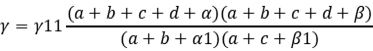  IC-2SD=E(IC)-2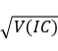  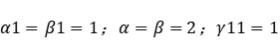 | 1.According to the strength of the signal  ①No signal(-):IC-2SD≤0;  ②Weak signal(+): 0＜IC-2SD≤1.5;  ③Medium signal(++): 1.5＜IC-2SD≤3;  ④Strong signal(+++): IC-2SD＞3  2.According to whether there is signal or not  a≥3, IC-2SD＞0 |
| **EBGM** | 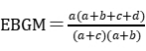  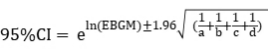 | EBGM_05_＞2 |

Equation: a, number of reports containing both the target drug and target adverse drug reaction; b, number of reports containing other adverse drug reaction of the target drug; c, number of reports containing the target adverse drug reaction of other drugs; d, number of reports containing other drugs and other adverse drug reactions. 95%Cl, 95% confidence interval; χ2, chi-squared; IC, information component; E(IC), the IC expectations; V(IC), the variance of IC; EBGM, empirical Bayesian geometric mean; EBGM_05_, the lower limit of 95% Cl of EBGM.

Supplementary Table 2. Signal detection for GLP-1 RAs-associated metabolic and nutritional adverse events at HLT level.

| **High level term** | **Drug** | **The report number** | **ROR (95% CI)** | **PRR (95% CI)** | **EBGM (EBGM05)** | **IC (IC025)** |
| --- | --- | --- | --- | --- | --- | --- |
| Appetite disorders |  |  |  |  |  |  |
|  | Semaglutide | 1,721 | 7.39 (7.04) | 7.18 (6.85) | 7.09 (6.81) | 0.35 (2.75) |
|  | Exenatide | 7,982 | 6.75 (6.59) | 6.57 (6.42) | 6.38 (6.26） | 0.37 (2.64) |
|  | Liraglutide | 1,523 | 5.77 (5.48) | 5.64 (5.37) | 5.61 (5.38) | 0.40 (2.41) |
|  | Tirzepatide | 722 | 5.20 (4.83) | 5.10 (4.74) | 5.00 (4.70) | 0.43 (2.20) |
|  | Dulaglutide | 2,783 | 4.56 (4.39) | 4.48 (4.32) | 4.42 (4.29) | 0.47 (2.09) |
|  | Albiglutide | 145 | 1.45 (1.23) | 1.45 (1.23) | 1.45 (1.26) | 1.88 (0.29) |
|  | Lixisenatide | 1 | - | - | 0.84 (0.16) | - |
| General nutritional disorders NEC |  |  |  |  |  |  |
|  | Liraglutide | 500 | 5.09 (4.06) | 5.06 (4.63) | 5.03 (4.67) | 0.43 (2.19) |
|  | Semaglutide | 374 | 4.38 (3.95) | 4.35 (3.93) | 4.32 (3.97) | 0.47 (1.95) |
|  | Tirzepatide | 121 | 2.38 (1.99) | 2.38 (1.99) | 2.36 (2.03) | 0.81 (0.96) |
|  | Dulaglutide | 528 | 2.17 (1.99) | 2.17 (1.99) | 2.16 (2.01) | 0.90 (0.98) |
|  | Exenatide | 502 | 1.16 (1.07) | 1.16 (1.06) | 1.16 (1.08) | 4.63 (0.09) |
|  | Albiglutide | 17 | 0.44 (0.27) | 0.44 (0.27) | 0.44 (0.29) | -0.84 (-1.83) |
|  | Lixisenatide | 1 | - | - | 2.11 (0.41) | - |
| Total fluid volume decreased |  |  |  |  |  |  |
|  | Semaglutide | 461 | 4.76 (4.34) | 4.72 (4.31) | 4.69 (4.34) | 0.45 (2.08) |
|  | Liraglutide | 387 | 3.12 (2.82) | 3.11 (2.81) | 3.10 (2.85) | 0.61 (1.48) |
|  | Tirzepatide | 159 | 2.99 (2.56) | 2.98 (2.55) | 2.95 (2.59) | 0.64 (1.31) |
|  | Dulaglutide | 614 | 2.30 (2.12) | 2.29 (2.11) | 2.28 (2.13) | 0.84 (1.07) |
|  | Exenatide | 512 | 0.87 (0.79) | 0.87 (0.79) | 0.87 (0.81) | -4.83 (-0.33) |
|  | Albiglutide | 24 | 0.55 (0.37) | 0.55 (0.37) | 0.55 (0.40) | -1.17 (-1.40) |
|  | Lixisenatide | 1 | - | - | 2.06 (0.40) | - |
| Diabetic complications NEC |  |  |  |  |  |  |
|  | Semaglutide | 177 | 5.79 (4.99) | 5.78 (4.98) | 5.72 (5.05) | 0.40 (2.26) |
|  | Liraglutide | 120 | 4.01 (3.35) | 4.01 (3.35) | 3.99 (3.43) | 0.50 (1.70) |
|  | Dulaglutide | 233 | 3.08 (2.71) | 3.08 (2.71) | 3.06 (2.74) | 0.62 (1.41) |
|  | Tirzepatide | 26 | 2.15 (1.46) | 2.15 (1.46) | 2.13 (1.54) | 0.91 (0.48) |
|  | Exenatide | 108 | 0.81 (0.67) | 0.81 (0.67) | 0.81 (0.69) | -3.22(-0.59) |
|  | Albiglutide | 4 | 0.33 (0.12) | 0.33 (0.12) | 0.33 (0.15) | -0.63(-2.68) |
|  | Lixisenatide | 2 | - | - | 13.16 (4.11) | - |
| Hypoglycaemic conditions NEC |  |  |  |  |  |  |
|  | Lixisenatide | 3 | 17.14 (5.49) | 16.94 (5.50) | 16.94 (6.53) | 0.24 (0.31) |
|  | Tirzepatide | 84 | 5.35 (4.31) | 5.34 (4.30) | 5.23 (4.36) | 0.42 (2.00) |
|  | Liraglutide | 164 | 3.66 (3.14) | 3.65 (3.14) | 3.64 (3.20) | 0.54 (1.62) |
|  | Dulaglutide | 324 | 3.34 (2.99) | 3.33 (2.99) | 3.30 (3.01) | 0.58 (1.55) |
|  | Exenatide | 718 | 3.29 (3.05) | 3.28 (3.05) | 3.24 (3.05) | 0.59 (1.58) |
|  | Semaglutide | 109 | 3.06 (2.53) | 3.05 (2.53) | 3.04 (2.60) | 0.62 (1.30) |
|  | Albiglutide | 22 | 1.40 (0.92) | 1.40 (0.92) | 1.39 (0.98) | 2.08 (-0.15) |
| Hyperglycaemic conditions NEC |  |  |  |  |  |  |
|  | Lixisenatide | 4 | 24.24 (9.02) | 23.87 (9.03) | 23.86 (10.44) | 0.22 (0.79) |
|  | Semaglutide | 64 | 1.84 (1.44) | 1.84 (1.44) | 1.83 (1.49) | 1.14 (0.50) |
|  | Liraglutide | 71 | 1.72 (1.37) | 1.72 (1.37) | 1.72 (1.42) | 1.28 (0.43) |
|  | Exenatide | 302 | 1.53 (1.37) | 1.53 (1.37) | 1.53 (1.39) | 1.64 (0.44) |
|  | Dulaglutide | 136 | 1.51 (1.28) | 1.51 (1.28) | 1.51 (1.31) | 1.68 (0.34) |
|  | Albiglutide | 8 | 0.55 (0.28) | 0.56 (0.28) | 0.56 (0.31) | -1.18 (-1.74) |
|  | Tirzepatide | 10 | 0.54 (0.29) | 0.54 (0.29) | 0.54 (0.32) | -1.14 (-1.69) |
| Diabetes mellitus (incl subtypes) |  |  |  |  |  |  |
|  | Liraglutide | 190 | 1.53 (1.33) | 1.53 (1.33) | 1.53 (1.36) | 1.63 (0.40) |
|  | Exenatide | 834 | 1.39 (1.30) | 1.39 (1.30) | 1.39 (1.31) | 0.00 (0.37) |
|  | Dulaglutide | 352 | 1.36 (1.22) | 1.36 (1.22) | 1.36 (1.24) | 2.28 (0.28) |
|  | Semaglutide | 124 | 1.28 (1.07) | 1.28 (1.07) | 1.28 (1.10) | 2.83 (0.09) |
|  | Albiglutide | 24 | 0.57 (0.38) | 0.57 (0.39) | 0.57 (0.41) | -1.25 (-1.35) |
|  | Tirzepatide | 9 | 0.16 (0.08) | 0.16 (0.08) | 0.16 (0.09) | -0.38 (-3.44) |
|  | Lixisenatide | 2 | - | - | 4.27 (1.33) | - |

PRR, the proportional reporting ratio; ROR, the reporting odds ratio; IC, the information component; EBGM, the empirical Bayes geometric mean; CI, confidence interval; 95% CI, two‐ sided for ROR, c2, chi-squared; IC025 and EBGM05 lower one‐sided for IC and EBGM.

Supplementary Table 3. Differences in detailed clinical characteristics between severe and non-severe reports.

| **Drug** |  | **Serious cases** | | **Non-serious cases** | **Statistic** | **p -value** |
| --- | --- | --- | --- | --- | --- | --- |
| **Exenatide** |  | |  |  |  |  |
|  | Gender, n (%) | |  |  |  |  |
|  | Male | | 852 (23.32) | 2,802 (76.68) | 13.776^b^ | <0.001^a^ |
|  | Female | | 1,351 (20.19) | 5,340 (79.81) |  |  |
|  | Age, years (median, IQR) | | 60 (52–68) | 62 (54–69) | -2.534^d^ | 0.011^c^ |
|  | Weight, kg (median, IQR) | | 94.43 (81.63–112.49) | 96.6 (83.01–113.40) | -2.415^d^ | 0.016^c^ |
|  | Types of AEs, n (%) | |  |  |  |  |
|  | Decreased Appetite | | 804 (29.46) | 6,196 (69.60) | 1404.467^b^ | <0.001^a^ |
|  | Hypoglycaemia | | 268 (9.82) | 422 (4.74) | 96.587^b^ | <0.001^a^ |
|  | Diabetes Mellitus Inadequate Control | | 171 (6.27) | 352 (3.95) | 22.023^b^ | <0.001^a^ |
|  | Weight Fluctuation | | 61 (2.24) | 184 (2.07) | 0.287^b^ | 0.592^a^ |
|  | Increased Appetite | | 46 (1.69) | 501 (5.63) | 72.433^b^ | <0.001^a^ |
|  | Food Craving | | 14 (0.51) | 126 (1.42) | 14.303^b^ | <0.001^a^ |
|  | Appetite Disorder | | 9 (0.33) | 82 (0.92) | 9.409^b^ | 0.002^a^ |
|  | Shock Hypoglycaemic | | 8 (0.29) | 1 (0.01) | 17.978^b^ | <0.001^a^ |
|  | Food Aversion | | 6 (0.22) | 53 (0.60) | 5.836^b^ | 0.016^a^ |
|  | Hypoglycaemia Unawareness | | 6 (0.22) | 8 (0.09) | 1.954^b^ | 0.162^a^ |
|  | Dawn Phenomenon | | 2 (0.07) | 4 (0.04) | 0.008^b^ | 0.929^a^ |
|  | Acquired Mixed Hyperlipidaemia | | 1 (0.04) | 2 (0.02) | - | 0.552^e^ |
|  | Somogyi Phenomenon | | 1 (0.04) | 2 (0.02) | - | 0.552^e^ |
|  |  | |  |  |  |  |
| **Liraglutide** |  | |  |  |  |  |
|  | Gender, n (%) | |  |  |  |  |
|  | Male | | 407 (46.09) | 476 (53.91) | 25.577^b^ | <0.001^a^ |
|  | Female | | 699 (36.07) | 1,239 (63.93) |  |  |
|  | Age, years (median, IQR) | | 60 (50–68) | 60 (52–68) | -0.565^d^ | 0.572^c^ |
|  | Weight, kg (median, IQR) | | 89 (73–106.6) | 91.2 (79.6–112.45) | -1.773^d^ | 0.076^c^ |
|  | Types of AEs, n (%) | |  |  |  |  |
|  | Dehydration | | 318 (23.93) | 63 (3.33) | 219.53^b^ | <0.001^a^ |
|  | Decreased Appetite | | 247 (18.59) | 927 (48.97) | 311.248^b^ | <0.001^a^ |
|  | Hypoglycaemia | | 106 (7.98) | 56 (2.96) | 41.169^b^ | <0.001^a^ |
|  | Diabetic Ketoacidosis | | 104 (7.83) | 0 (0.00) | 153.076^b^ | <0.001^a^ |
|  | Diabetes Mellitus Inadequate Control | | 81 (6.09) | 73 (3.86) | 8.597^b^ | 0.003^a^ |
|  | Weight Loss Poor | | 45 (3.39) | 322 (17.01) | 143.595^b^ | <0.001^a^ |
|  | Increased Appetite | | 16 (1.20) | 188 (9.93) | 100.282^b^ | <0.001^a^ |
|  | Food Craving | | 13 (0.98) | 50 (2.64) | 11.266^b^ | <0.001^a^ |
|  | Feeding Disorder | | 11 (0.83) | 44 (2.32) | 10.424^b^ | 0.001^a^ |
|  | Appetite Disorder | | 10 (0.75) | 22 (1.16) | 1.333^b^ | 0.248^a^ |
|  | Hyperlipasaemia | | 9 (0.68) | 0 (0.00) | 10.539^b^ | 0.001^a^ |
|  | Food Aversion | | 6 (0.45) | 9 (0.48) | 0.010^b^ | 0.922^a^ |
|  | Diabetic Metabolic Decompensation | | 6 (0.45) | 0 (0.00) | 6.306^b^ | 0.012^a^ |
|  | Ketosis | | 4 (0.30) | 1 (0.05) | 1.708^b^ | 0.191^a^ |
|  | Hyperamylasaemia | | 4 (0.30) | 0 (0.00) | 3.535^b^ | 0.060^a^ |
|  | Insulin Resistance | | 3 (0.23) | 4 (0.21) | 0.000^b^ | 1.000^a^ |
|  | Lack Of Satiety | | 1 (0.08) | 13 (0.69) | 6.749^b^ | 0.009^a^ |
|  |  | |  |  |  |  |
| **Albiglutide** |  | |  |  |  |  |
|  | Gender, n (%) | |  |  |  |  |
|  | Male | | 32 (37.65) | 53 (62.35) | 0.595^b^ | 0.441^a^ |
|  | Female | | 48 (32.65) | 99 (67.35) |  |  |
|  | Age, years (median, IQR) | | 61.5(53.25–69) | 59 (53–65) | -1.465^d^ | 0.143^c^ |
|  | Weight, kg (median, IQR) | | 89.63 (78.47–116.66) | 88.67 (73.47–109.34) | -0.674^d^ | 0.527^c^ |
|  | Types of AEs, n (%) | |  |  |  |  |
|  | Diabetes Mellitus Inadequate Control | | 23 (20.54) | 0 (0.00) | 222.988^b^ | <0.001^a^ |
|  |  | |  |  |  |  |
| **Dulaglutide** |  | |  |  |  |  |
|  | Gender, n (%) | |  |  |  |  |
|  | Male | | 745 (42.50) | 1,008 (57.50) | 37.710^b^ | <0.001^a^ |
|  | Female | | 866 (33.33) | 1,732 (66.67) |  |  |
|  | Age, years (median, IQR) | | 62 (51.75–72) | 63 (55–71) | -1.237^d^ | 0.216^c^ |
|  | Weight, kg (median, IQR) | | 85.00 (70.39–101.11) | 96.15 (77.10–115.47) | -2.940^d^ | 0.003^c^ |
|  | Types of AEs, n (%) | |  |  |  |  |
|  | Dehydration | | 434 (20.90) | 171 (5.12) | 321.4^b^ | <0.001^a^ |
|  | Decreased Appetite | | 430 (20.70) | 1,901 (56.90) | 684.54^b^ | <0.001^a^ |
|  | Diabetic Ketoacidosis | | 175 (8.43) | 0 (0.00) | 290.896^b^ | <0.001^a^ |
|  | Hypoglycaemia | | 150 (7.22) | 165 (4.94) | 12.194^b^ | <0.001^a^ |
|  | Diabetes Mellitus Inadequate Control | | 126 (6.07) | 130 (3.89) | 13.464^b^ | <0.001^a^ |
|  | Feeding Disorder | | 100 (4.81) | 265 (7.93) | 19.807^b^ | <0.001^a^ |
|  | Hypophagia | | 65 (3.13) | 115 (3.44) | 0.39^b^ | 0.533^a^ |
|  | Ketoacidosis | | 58 (2.79) | 0 (0.00) | 94.307^b^ | <0.001^a^ |
|  | Diabetic Complication | | 21 (1.01) | 6 (0.18) | 17.858^b^ | <0.001^a^ |
|  | Marasmus | | 17 (0.82) | 0 (0.00) | 27.432^b^ | <0.001^a^ |
|  | Increased Appetite | | 14 (0.67) | 130 (3.89) | 51.233^b^ | <0.001^a^ |
|  | Ketosis | | 13 (0.63) | 1 (0.03) | 17.651^b^ | <0.001^a^ |
|  | Hyperglycaemic Hyperosmolar Nonketotic Syndrome | | 12 (0.58) | 0 (0.00) | 16.820^b^ | <0.001^a^ |
|  | Fluid Intake Reduced | | 10 (0.48) | 30 (0.90) | 3.031^b^ | 0.082^a^ |
|  | Insulin Resistance | | 10 (0.48) | 17 (0.51) | 0.019^b^ | 0.889^a^ |
|  | Overweight | | 8 (0.39) | 41 (1.23) | 10.132^b^ | 0.001^a^ |
|  | Appetite Disorder | | 5 (0.24) | 51 (1.53) | 20.699^b^ | <0.001^a^ |
|  | Diabetic Ketosis | | 4 (0.19) | 0 (0.00) | 4.093^b^ | 0.043^a^ |
|  | Food Aversion | | 3 (0.14) | 9 (0.27) | 0.428^b^ | 0.513^a^ |
|  | Food Craving | | 2 (0.10) | 42 (1.26) | 21.425^b^ | 0.001^a^ |
|  | Starvation | | 1 (0.05) | 5 (0.15) | 0.452^b^ | 0.501^a^ |
|  | Lack Of Satiety | | 1 (0.05) | 4 (0.12) | 0.147^b^ | 0.701^a^ |
|  | Shock Hypoglycaemic | | 1 (0.05) | 3 (0.09) | 0.001^b^ | 0.973^a^ |
|  | Dawn Phenomenon | | 1 (0.05) | 2 (0.06) | 0.000^b^ | 1.000^a^ |
|  |  | |  |  |  |  |
| **Semaglutide** |  | |  |  |  |  |
|  | Gender, n (%) | |  |  |  |  |
|  | Male | | 471 (43.94) | 601 (56.06) | 2.587^b^ | 0.108^a^ |
|  | Female | | 715 (40.86) | 1,035 (59.14) |  |  |
|  | Age, years (median, IQR) | | 61 (51–71) | 65 (57–72) | -5.475^d^ | <0.001^c^ |
|  | Weight, kg (median, IQR) | | 88 (74–103) | 93 (77.6–111) | -2.357^d^ | 0.018^c^ |
|  | Types of AEs, n (%) | |  |  |  |  |
|  | Dehydration | | 370 (25.10) | 81 (4.42) | 296.307^b^ | <0.001^a^ |
|  | Decreased Appetite | | 362 (24.56) | 1,015 (55.43) | 320.284^b^ | <0.001^a^ |
|  | Diabetic Ketoacidosis | | 138 (9.36) | 2 (0.11) | 172.356^b^ | <0.001^a^ |
|  | Hypoglycaemia | | 68 (4.61) | 36 (1.97) | 18.776^b^ | <0.001^a^ |
|  | Hypophagia | | 49 (3.32) | 41 (2.24) | 3.630^b^ | 0.057^a^ |
|  | Diabetes Mellitus Inadequate Control | | 41 (2.78) | 61 (3.33) | 0.826^b^ | 0.363^a^ |
|  | Feeding Disorder | | 37 (2.51) | 61 (3.33) | 1.915^b^ | 0.166^a^ |
|  | Euglycaemic Diabetic Ketoacidosis | | 32 (2.17) | 0 (0.00) | 40.139^b^ | <0.001^a^ |
|  | Ketoacidosis | | 28 (1.90) | 0 (0.00) | 35.079^b^ | <0.001^a^ |
|  | Increased Appetite | | 19 (1.23) | 129 (7.05) | 63.257^b^ | <0.001^a^ |
|  | Abnormal Loss Of Weight | | 15 (1.02) | 15 (0.82) | 0.357^b^ | 0.55^a^ |
|  | Weight Loss Poor | | 11 (0.75) | 150 (8.19) | 97.699^b^ | <0.001^a^ |
|  | Food Aversion | | 10 (0.68) | 30 (1.64) | 6.295^b^ | 0.012^a^ |
|  | Fluid Intake Reduced | | 8 (0.54) | 9 (0.49) | 0.042^b^ | 0.838^a^ |
|  | Ketosis | | 8 (0.54) | 0 (0.00) | 7.841^b^ | 0.005^a^ |
|  | Food Craving | | 5 (0.34) | 46 (2.51) | 25.382^b^ | <0.001^a^ |
|  | Insulin Resistance | | 5 (0.34) | 8 (0.44) | 0.199^b^ | 0.656^a^ |
|  | Starvation Ketoacidosis | | 4 (0.27) | 0 (0.00) | 2.983^b^ | 0.084^a^ |
|  | Appetite Disorder | | 2 (0.14) | 32 (1.75) | 20.841^b^ | <0.001^a^ |
|  | Hyperphagia | | 2 (0.14) | 11 (0.60) | 4.508^b^ | 0.034^a^ |
|  | Postprandial Hypoglycaemia | | 1 (0.08) | 2 (0.11) | 0.000^b^ | 1.000^a^ |
|  | Lack Of Satiety | | 0 (0.00) | 7 (0.38) | 3.983^b^ | 0.046^a^ |
|  |  | |  |  |  |  |
| **Tirzepatide** |  | |  |  |  |  |
|  | Gender, n (%) | |  |  |  |  |
|  | Male | | 55 (24.12) | 173 (75.88) | 11.664^b^ | <0.001^a^ |
|  | Female | | 99 (14.37) | 590 (85.63) |  |  |
|  | Age, years (median, IQR) | | 53 (44–61) | 53 (44–62) | -0.505^d^ | 0.614^c^ |
|  | Weight, kg (median, IQR) | | 103.75 (85.5–120.25) | 107.8 (69.75–135.88) | -0.454^d^ | 0.65^c^ |
|  | Types of AEs, n (%) | |  |  |  |  |
|  | Dehydration | | 70 (32.86) | 83 (8.50) | 92.535^b^ | <0.001^a^ |
|  | Decreased Appetite | | 31 (14.55) | 519 (53.18) | 104.911^b^ | <0.001^a^ |
|  | Feeding Disorder | | 17 (7.98) | 57 (5.84) | 1.373^b^ | 0.241^a^ |
|  | Hypoglycaemia | | 16 (7.51) | 67 (6.86) | 0.113^b^ | 0.737^a^ |
|  | Increased Appetite | | 4 (1.88) | 90 (9.22) | 12.95^b^ | <0.001^a^ |
|  | Fluid Intake Reduced | | 4 (1.88) | 7 (0.72) | 1.460^b^ | 0.227^a^ |
|  | Food Craving | | 0 (0.00) | 30 (3.07) | 5.525^b^ | 0.019^a^ |
|  | Appetite Disorder | | 0 (0.00) | 19 (1.95) | 3.067^b^ | 0.08^a^ |
|  | Starvation | | 0 (0.00) | 17 (1.74) | 2.629^b^ | 0.105^a^ |
|  | Food Aversion | | 0 (0.00) | 7 (0.72) | 0.556^b^ | 0.456^a^ |
|  | Insulin Resistance | | 0 (0.00) | 5 (0.51) | - | 0.592^e^ |
|  | Lack Of Satiety | | 0 (0.00) | 3 (0.31) | - | 1^e^ |

^a^Proportions were compared using Pearson χ^2^ test.

^b^The χ^2^ statistic of the Pearson chi-square test.

^c^Mann-Whitney U test.

^d^The Z statistic of the Mann-Whitney U test.

^e^Fisher's exact test.

p-value < 0.05 were considered statistically significant.

Supplementary Table 4. Detailed clinical characterization of differences in whether or not appetite disorders occurs.

| **Drug** |  | **Appetite disorders** | **Non-appetite disorders** | **Statistic** | ***p* -value** |
| --- | --- | --- | --- | --- | --- |
| **Exenatide** |  |  |  |  |  |
|  | Gender, *n* (%) |  |  |  |  |
|  | Male | 2,575 (70.47) | 1,079 (29.53) | 21.524 | <0.001 |
|  | Female | 4,998 (74.70) | 1,693 (25.30) |  |  |
|  | Age, years (median, IQR) | 61 (54–67) | 60 (52–68) | -1.903 | 0.057 |
|  | Weight, kg (median, IQR) | 96.16 (83–131.09) | 95.25 (81.65–112.5) | -1.676 | 0.094 |
| **Liraglutide** |  |  |  |  |  |
|  | Gender, *n* (%) |  |  |  |  |
|  | Male | 436 (49.38) | 447 (50.62) | 2.333 | 0.127 |
|  | Female | 1,017 (52.48) | 921 (47.52) |  |  |
|  | Age, years (median, IQR) | 61 (53–69) | 59 (50–67) | -3.918 | <0.001 |
|  | Weight, kg (median, IQR) | 85 (70.3–104.3) | 90.9 (77–110) | -2.878 | 0.004 |
| **Dulaglutide** |  |  |  |  |  |
|  | Gender, *n* (%) |  |  |  |  |
|  | Male | 928 (52.94) | 825 (47.06) | 7.277 | 0.007 |
|  | Female | 1,483 (57.08) | 1,115 (42.92) |  |  |
|  | Age, years (median, IQR) | 64 (55–72) | 62 (51–71) | -3.832 | <0.001 |
|  | Weight, kg (median, IQR) | 83.2 (67.98–102) | 89.6 (73.69–104.30) | -1.717 | 0.086 |
| **Semaglutide** |  |  |  |  |  |
|  | Gender, *n* (%) |  |  |  |  |
|  | Male | 654 (61.01) | 418 (38.99) | 6.234 | 0.013 |
|  | Female | 984 (56.23) | 766 (43.77) |  |  |
|  | Age, years (median, IQR) | 65 (56–73) | 61 (51–70) | -5.979 | <0.001 |
|  | Weight, kg (median, IQR) | 88 (73.25–105.58) | 89.15 (76.53–104) | -0.949 | 0.343 |
| **Tirzepatide** |  |  |  |  |  |
|  | Gender, *n* (%) |  |  |  |  |
|  | Male | 138 (60.53) | 90 (39.47) | 7.418 | 0.006 |
|  | Female | 484 (70.25) | 205 (29.75) |  |  |
|  | Age, years (median, IQR) | 53 (44–62) | 52.5 (44.75–62) | -0.346 | 0.729 |
|  | Weight, kg (median, IQR) | 99.8 (80.1–131.1) | 104 (87.75–131) | -0.640 | 0.562 |

*P* -value < 0.05 were considered statistically significant.
