# Supplementary material for: Pharmacovigilance study of GLP-1 receptor agonists for metabolic and nutritional adverse events
Source: Front Pharmacol. 2024 Jul 8;15:1416985. doi: 10.3389/fphar.2024.1416985 (PMC11260617; doi:10.3389/fphar.2024.1416985)
Supplement: Supplementary file 2 [file Table2.DOCX]

Supplementary Material

Pharmacovigilance Study of GLP-1 Receptor Agonists for Metabolic and Nutritional Adverse Events

**Long He*****, Qiuyu Li*****, Yongfeng Yang, Jiahao Li, Wei Luo, Yilan Huang, Xiaoyan Zhong**

*** Correspondence:** Yilan Huang: [hyl3160131@126.com](mailto:hyl3160131@126.com), Xiaoyan Zhong: [312556662@qq.com](mailto:312556662@qq.com)

# Supplementary Data

DRUG[unique(inter.demo$primaryid)]->inter.drug

if(!missing(drug)) {

target.drug <-inter.drug[(DRUGNAME %in% drug | prod_ai %in% drug)&( ROLE_COD %in% drugEffect)]

target.drug <- as.numeric(NROW(target.drug))

df1 <- data.frame(

Name = c("目标药物数据量"),

number= c( target.drug)

)

}

inter.drug <-as.numeric(NROW(inter.drug))

rm(DRUG)

#

OUTC[unique(inter.demo$primaryid)]->inter.outc

inter.outc<- as.numeric(NROW(inter.outc))

rm(OUTC)

#

INDI[unique(inter.demo$primaryid)]->inter.indi

inter.indi<- as.numeric(NROW(inter.indi))

rm(INDI)

#

REAC[unique(inter.demo$primaryid)]->inter.reac

inter.reac<- as.numeric(NROW(inter.reac))

rm(REAC)

#

RPSR[unique(inter.demo$primaryid)]->inter.rpsr

inter.rpsr<- as.numeric(NROW(inter.rpsr))

rm(RPSR)

#

THER[unique(inter.demo$primaryid)]->inter.ther

inter.ther<- as.numeric(NROW(inter.ther))

rm(THER)

#

DEMO[unique(inter.demo$primaryid)]->inter.demo

inter.demo<- as.numeric(NROW(inter.demo))

rm(DEMO)

### 创建数据表格

df2 <- data.frame(

Name = c("去重前DEMO数据量", "去重后DEMO数据量", "去重后DRUG数据量","去重后reac数据量",

"去重后THER数据量","去重后RPSR数据量","去重后THER数据量","去重后OUTC数据量"),

number= c(rawDEMO,inter.demo,inter.drug,inter.reac,inter.ther,inter.rpsr,inter.ther,inter.outc)

)

df2 <- rbind(df2,df1)

write.xlsx(df2,file=file.path(path,'流程图数据.xlsx'))
